# A new species and two new combinations of *Monolophus* (Zingiberaceae) from Indo-Burma

**DOI:** 10.3897/phytokeys.138.39217

**Published:** 2020-01-10

**Authors:** Hong-Bo Ding, Bin Yang, Mya Bhone Maw, Pyae Pyae Win, Yun-Hong Tan

**Affiliations:** 1 Southeast Asia Biodiversity Research Institute, Chinese Academy of Sciences & Center for Integrative Conservation, Xishuangbanna Tropical Botanical Garden, Chinese Academy of Sciences, Menglun, Mengla, Yunnan 666303, China; 2 Center of Conservation Biology, Core Botanical Gardens, Chinese Academy of Sciences, Menglun, Mengla,Yunnan 666303, China; 3 Forest Research Institute, Forest Department, Ministry of Environmental Conservation and Forestry, Yezin, Nay Pyi Taw 05282, Myanmar

**Keywords:** *Caulokaempferia phokhamii*, *Caulokaempferia wongsuwaniae*, Kachin State, Putao District

## Abstract

*Monolophus odontochilus* Y.H.Tan & H.B.Ding, a new species from Northern Myanmar, is described and illustrated. The new species is morphologically similar to *M. linearis*, but differs by having elliptic to oblong leaves (vs. linear-lanceolate to lanceolate), bilobed ligules (vs. entire), purely white corolla (vs. pinkish white), semi-orbicular crenate labellum (vs. trilobed). In addition, a diagnostic key to the new species of *Monolophus* and its closely related non-yellow flowered species is provided. New combinations are proposed here for *Caulokaempferia phokhamii* Picheans. & Douangde. and *C. wongsuwaniae* Picheans. & Douangde. from Laos.

## Introduction

The genus *Monolophus* was first described by Wallich (1832) and included three species with references to earlier publications in 1820, 1829; and was recognised by Endlicher (1837), followed by Steudel (1841), Horaninov (1862), Pfeiffer (1874), Wu and Chen (1978). But for a very long time it was treated under *Caulokaempferia* K. Larsen (1964). Recently, the genus *Monolophus* was reinstated by Mood et al. (2014) with 22 new combinations. So far, the genus consists of 33 species (Larsen and Smith 1972; Larsen 1973, 2002; Larsen et al. 2004; Larsen and Jenjittikul 2004; Suksathan and Triboun 2004; Picheansoonthon and Mokkamul 2006; Ngamriabsakul 2008; Picheansoonthon et al. 2008; Picheansoonthon and Koonterm 2008; Tiyaworanant 2010; Chaturvedi et al. 2012; Roy and Barbhuiya 2013; Intharapichai et al. 2014; Mood et al. 2014; Phokham et al. 2015a, b; Sangnark et al. 2016; Veldkamp 2016; Bhaumik et al. 2017; Barbhuiya et al. 2018; Douangdeuane et al. 2019) from the Himalayas to South East Asia. There are two distinct groups, the yellow-flowered species (~28 taxa) that are distributed in Thailand and adjacent countries and the other non-yellow-flowered species (purple, red-purple, pink and white) (~5 taxa) which are localized in Eastern Himalaya (Bhaumik et al. 2017).

During our field work from May to June in 2018, some interesting specimens of *Monolophus* were found in Putao, Kachin state. Based on the detailed examination of the morphological characters of our material and morphologically similar species, we draw a conclusion that those specimens of *Monolophus* collected in Myanmar belong to a species new to science, *Monolophus odontochilus* Y.H.Tan & H.B.Ding, which is described here along with illustrations.

## Material and methods

Measurements and morphological character assessments of the new species have been examined based on fresh materials and dried specimens. It has been compared with the morphologically similar species by affinities inferred using descriptions (Wallich 1820, 1832; Chaturvedi et al. 2012; Roy and Barbhuiya 2013) and type specimens in herbaria (K, L, E). Protologues and images of type specimens and dried herbarium specimens were gathered from JSTOR Global Plants (http://plants.jstor.org).

## Taxonomic treatment

### Monolophus odontochilus

Taxon classificationPlantaeZingiberalesZingiberaceae

Y.H.Tan & H.B.Ding sp. nov.

84E09738-621C-516C-9E79-13F88DB1D982

urn:lsid:ipni.org:names:77204203-1

Figure 1

#### Diagnosis.

*Monolophus odontochilus* is morphologically similar to *M. linearis* (Wall.) Wall. from India in having white flowers with yellow blotch at the base of the labellum, but differs by having elliptic to oblong leaves (vs. linear-lanceolate to lanceolate leaves), bilobed ligules (vs. entire ligules), semi-orbicular crenate labellum (vs. trilobed labellum) and purely white corolla (vs. pinkish white corolla).

#### Type.

Myanmar. Kachin State: Putao District, Gathu Village. 27°29'07.23"N, 97°58'19.49"E, 643 m, 01 June 2018, *Y.H.Tan*, *B.Yang*, *H.B.Ding*, *X.D.Zeng*, *M.B.Maw and H.L.Neing M3886* (holotype: HITBC!, isotypes: RAF!).

#### Description.

Perennial herbs, rhizome short; pseudostem erect, leafy 50–70 cm long. ***Leaves*** 5–13, sub-sessile, elliptic to oblong, 10.2–14 × 3.0–5.0 cm, base cuneate, apex caudate to 2.0 cm long, margins entire, adaxial surface dark green, glabrous, abaxially light green, pubescent; ***ligule*** membranous, 6–8 mm long, hairy, apex unequally bilobed. ***Inflorescences*** terminal, 5–7 cm long, rachis glabrous; ***flowers*** white with yellow blotch at the base of the labellum; ***bracts*** 4–6, along rachis, distichous, oblong, 3.5–4.0 × 1.0–1.3 cm, greenish, outer surface pubescent, base truncate, apex cuspidate to 0.8 cm, 2-flowered; ***bracteoles*** membranous, lanceolate, ca. 1.5 × 0.8 cm, pubescent, rounded; ***calyx*** tubular, 1.3–1.4 × 0.3–0.4 cm, pubescent, greenish, split 6–7 mm down one side, apex acute; ***floral tube*** 4.3–4.5 cm, ca. 3.5 mm wide at mouth, white, glabrous; ***dorsal corolla lobe*** lanceolate, 2.2–2.3 × 0.6–0.7 cm, white, glabrous, hooded at apex, apical cusp ca. 2 mm long; ***lateral corolla lobes*** similar to dorsal lobe, narrower, 2.0–2.1 × 0.3–0.4 cm, hooded at apex, apical cusp ca. 1 mm long; ***lateral staminodes*** elliptic, 1.5–1.6 × 0.9–1.0 cm, white, apex rounded; ***labellum*** semi-orbicular, 4.4–4.8 × 3.2–3.6 cm, white with yellow spot at base, margin crenate; ***anther*** 4–5 mm long; ***anther crest*** flabellate, 2–3 × 4–5 mm, white, apex entire; ***stigma*** funnel-shaped, inserted between anther sac, margin raised on both ends, ciliate; ***ovary*** oblong, 3–4 mm long, pubescent, 3-locular.

**Figure 1.**
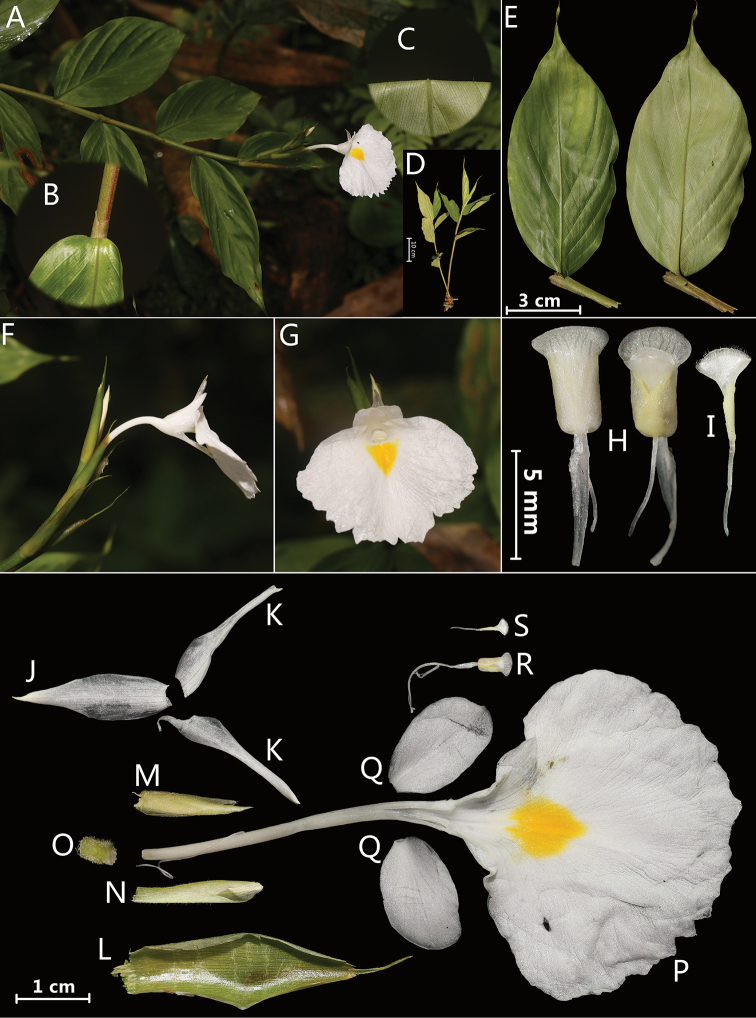
*Monolophus odontochilus* Y.H. Tan & H.B. Ding, sp. nov. **A** habitat **B** ligule **C** leaf blade abaxially **D** habit **E** single leaf (adaxially and abaxially) **F** flower (side view) **G** flower (front view) **H** anther with stigma and crest (front and back view) **I** stigma **J** dorsal corolla lobe **K** lateral corolla lobes **L** bracts **M** bracteoles and young flower **N** calyx **O** ovary **P** labellum and floral tube **Q** lateral staminodes **R** stamen and stigma **S** ovary with style. Photographed by H.B.Ding

#### Phenology.

Flowering in May to June. Fruit not seen.

#### Etymology.

The species epithet ‘*odontochilus*’ refers to the crenate labellum.

#### Distribution and habitat.

*Monolophus odontochilus* is endemic to Kachin State, Northern Myanmar, only known from its type locality, Gathu Village, Putao District. It grows in humid environments or along streams of tropical rain forest at an elevation of 550–750 m.

#### Conservation status.

This new species appears to be restricted to a very moist habitat in Gathu Village, Putao District, Kachin State, Northern Myanmar. According to our observations in the field, the two known populations are composed of about 50 mature individuals in each. Overall, however, the species has been deemed to be Data Deficient (DD) following IUCN Red list Categories and Criteria (IUCN 2017). Further field surveys in northern Myanmar are needed to gain more information on its distribution.

#### Affinities.

*Monolophus odontochilus* is morphologically similar to *M. linearis* (Wall.) Wall. (Wallich 1820, 1832) in having white flowers with a yellow blotch at the base of the labellum. After comparison with specimens and descriptions in literature (Chaturvedi et al. 2012; Roy and Barbhuiya 2013), it was found that *M. odontochilus* can be distinguished from *M. linearis*, even on the basis of its vegetative characters: e.g. *Monolophus odontochilus* has unequally bilobed ligule (vs. entire ligule, respectively), longer calyx (1.3–1.4 cm vs. 0.8–0.9 cm, respectively), bigger lateral staminodes (1.5–1.6 × 0.9–1.0 cm vs. ca. 1.0 × 0.7 cm, respectively). *Monolophus odontochilus*, furthermore, differs in having elliptic to oblong leaves (10.2–14 × 3–5 cm), margin crenate labellum (non-trilobed) and purely white corolla. *Monolophu linearis* has linear lanceolate leaves (1.5–6.5 × 0.5–1 cm), trilobed labellum and pinkish white corolla. A diagnostic key to the new species of *Monolophus* and its closely related species is provided.

### A diagnostic key to the non-yellow flowered of *Monolophus* is given below

**Table:** 

1	Flowers completely pink	**2**
–	Flowers usually white, if corolla and lateral staminodes pink then labellum white	**3**
2	All leaves sessile, blade to 2.8 cm broad, ligule absent or indistinct	***M. suksathanii***
–	At least upper leaves petiolate, blade 2.5–4 cm broad, ligule distinct	***M. secundus***
3	Labellum completely white, without yellow blotch	***M. sikkimensis***
–	Labellum white with yellow blotch	**4**
4	Corolla completely white	***M. odontochilus***
–	Corolla pink to purple-pink	**5**
5	Leaf blades linear-lanceolate to lanceolate, labellum trilobed	***M. linearis***
–	Leaf blades oblong to oblong-elliptic, labellum entire	***M. arunachalensis***

### New combinations

Mood et al. (2014) have argued that the generic name *Caulokaempferia* is a superfluous name of genus *Monolophus*. A proposal to conserve *Caulokaempferia* by Intharapichai et al. (2014) has not yet been considered by the appropriate committee. Therefore, in our opinion, *Monolophus* is valid and the name *Caulokaempferia* must be rejected. Recently, Douangdeuane et al. (2019) described two new species under *Caulokaempferia* from Laos, which are transferred here to *Monolophus*.

#### Monolophus phokhamii

Taxon classificationPlantaeZingiberalesZingiberaceae

(Picheans. & Douangde.) Y.H.Tan & H.B.Ding comb. nov.

4C14D2E8-7A4F-5E2B-AF97-805E5A988838

urn:lsid:ipni.org:names:77204204-1

##### Basionym.

*Caulokaempferia phokhamii* Picheans. & Douangde. in Douangdeuane et al., Pak. J. Bot. 51(1): 235. 2019.

##### Type.

Lao PDR. Vientiane Province, Hin Herb District, Phou Meut-Phou Kiykon Forest Conservation, Ban Hoiuy Dokmai (Hoiuy Dokmai Waterfall), 18°42'58.20"N, 102°22'20.94"E, 270 m, 26 August 2014, *CP260814-1* (holotype: BK, isotypes: MSU).

#### Monolophus wongsuwaniae

Taxon classificationPlantaeZingiberalesZingiberaceae

(Picheans. & Douangde.) Y.H.Tan & H.B.Ding comb. nov.

2E9ADD78-3A14-537F-B169-4DFB3F1A3297

urn:lsid:ipni.org:names:77204205-1

##### Basionym.

*Caulokaempferia wongsuwaniae* Picheans. & Douangde. in Douangdeuane et al., Pak. J. Bot. 51(1): 237. 2019.

##### Type.

Lao PDR. Bolikhamxai Province, Mueang Tha Pabad District, Nam Tok Tad Mangkorn, 18°25'59.82"N, 103°12'32.64"E, 200 m, 20 September 2014, *CP200914-1* (holotype: BK, isotypes: MSU).

## Supplementary Material

XML Treatment for Monolophus odontochilus

XML Treatment for Monolophus phokhamii

XML Treatment for Monolophus wongsuwaniae
